# Cholinergic Control of Inflammation, Metabolic Dysfunction, and Cognitive Impairment in Obesity-Associated Disorders: Mechanisms and Novel Therapeutic Opportunities

**DOI:** 10.3389/fnins.2019.00263

**Published:** 2019-04-05

**Authors:** Eric H. Chang, Sangeeta S. Chavan, Valentin A. Pavlov

**Affiliations:** ^1^Center for Bioelectronic Medicine and Biomedical Sciences, Feinstein Institute for Medical Research, Northwell Health, Manhasset, NY, United States; ^2^Donald and Barbara Zucker School of Medicine at Hofstra/Northwell, Hempstead, NY, United States

**Keywords:** cholinergic, brain, vagus nerve, obesity, metabolic syndrome, inflammation, neuroinflammation, cognition

## Abstract

Obesity and obesity-associated disorders have become world-wide epidemics, substantially increasing the risk of debilitating morbidity and mortality. A characteristic feature of these disorders, which include the metabolic syndrome (MetS) and type 2 diabetes, is chronic low-grade inflammation stemming from metabolic and immune dysregulation. Inflammation in the CNS (neuroinflammation) and cognitive impairment have also been associated with obesity-driven disorders. The nervous system has a documented role in the regulation of metabolic homeostasis and immune function, and recent studies have indicated the important role of vagus nerve and brain cholinergic signaling in this context. In this review, we outline relevant aspects of this regulation with a specific focus on obesity-associated conditions. We outline accumulating preclinical evidence for the therapeutic efficacy of cholinergic stimulation in alleviating obesity-associated inflammation, neuroinflammation, and metabolic derangements. Recently demonstrated beneficial effects of galantamine, a centrally acting cholinergic drug and cognitive enhancer, in patients with MetS are also summarized. These studies provide a rationale for further therapeutic developments using pharmacological and bioelectronic cholinergic modulation for clinical benefit in obesity-associated disorders.

## Introduction

Obesity and obesity-related disorders have become prevalent conditions in our modern society, impacting over 1 billion people worldwide (Zimmet et al., 2001; Grundy, 2008; Aguilar et al., 2015; Kim et al., 2019). Obesity and the closely related metabolic syndrome (MetS) generate a substantial risk of developing type 2 diabetes, cardiovascular disease, and other debilitating and life-threatening diseases (Eckel et al., 2005; Grundy, 2008; van Dieren et al., 2010). Therefore, treating these conditions is of primary importance. Diet and exercise are key approaches, but in many cases these lifestyle modifications are either not sustainable or difficult to implement. Clearly, there is a considerable need for better understanding of the complex pathology underlying obesity-driven disorders and for strategizing novel therapeutic approaches. Obesity-related chronic low-grade inflammation provides an important link to metabolic derangements including insulin resistance in these disorders (Shoelson et al., 2007). In addition to peripheral inflammation, inflammation in the CNS (neuroinflammation), affecting the hypothalamus and other brain regions, has also been described and associated with cognitive impairment in the context of obesity (Guillemot-Legris and Muccioli, 2017). Multiple links between peripheral and brain alterations, involving inflammatory, metabolic, and neural components, have been identified in obesity-associated conditions (Schwartz and Porte, 2005; Pavlov and Tracey, 2012). Of note, the role of the nervous system in this relationship has been a specific focus of ongoing research. The nervous system and the brain regulate feeding behavior, energy intake and expenditure, and metabolic homeostasis (Pavlov and Tracey, 2012; Kaelberer et al., 2018). The vagus nerve (the tenth cranial nerve), which contains fibers carrying ascending sensory signals to the brain and descending motor signals to the visceral organs, is importantly involved in these regulatory processes (Pavlov et al., 2018).

The nervous system also communicates with the immune system (Dantzer, 2018; Pavlov et al., 2018), and research during the last 20 years has revealed the important role of the vagus nerve in this communication. A body of preclinical evidence has demonstrated that the vagus nerve regulates inflammatory responses within a physiological mechanism termed *the inflammatory reflex* (Tracey, 2002; Pavlov et al., 2003). Accordingly, electrical vagus nerve stimulation (VNS) has been shown to control the release of pro-inflammatory cytokines and aberrant inflammation in many conditions (Chavan et al., 2017; Pavlov and Tracey, 2017). The mechanisms of the inflammatory reflex, which will be discussed in more detail later, involve alpha7 nicotinic acetylcholine receptor (α7nAChR)-mediated signaling in its efferent arm. Cholinergic compounds, including α7nAChR agonists and centrally acting acetylcholinesterase inhibitors (AChE), have also been shown to alleviate inflammation and metabolic derangements in obesity and MetS (Pavlov and Tracey, 2012). One of these drugs, the centrally acting AChE inhibitor galantamine is in clinical use for counteracting cognitive impairment in Alzheimer’s disease (Hampel et al., 2018). A recent clinical trial revealed the anti-inflammatory and beneficial metabolic effects of galantamine in patients with MetS (Consolim-Colombo et al., 2017). Recent clinical studies also demonstrated the utility of bioelectronic VNS in rheumatoid arthritis and inflammatory bowel disease (IBD) – conditions characterized by immune and metabolic dysregulation (Bonaz et al., 2016; Koopman et al., 2016). Future applications of VNS in the arena of MetS and other obesity-driven disorders are feasible and of significant interest.

In this review, we briefly summarize the role of brain and the vagus nerve cholinergic signaling in the regulation of metabolic homeostasis and the role of the vagus nerve-based inflammatory reflex in controlling inflammation. We further point to important aspects of the relationship between inflammation, metabolic deterioration, neuroinflammation, and cognitive impairment in obesity-driven disorders. In this context, we elaborate on accumulating pre-clinical and clinical evidence for therapeutic benefit of stimulating brain and vagus nerve cholinergic signaling. We also consider the potential benefit of enhancing cholinergic signaling by centrally acting AChE inhibitors and VNS for counteracting cognitive deterioration in obesity-driven conditions.

## The Brain and Vagus Nerve in Metabolic Regulation and a Role for Cholinergic Signaling

One of the first indications that the brain regulates body metabolism came from studies performed by the 19th century French physiologist Claude Bernard. He reported that electrically stimulating the floor of the fourth ventricle in the brain increased circulating blood glucose and induced a type of transient diabetes, thus connecting the brain with glucose and diabetes (Bernard, 1855). This intriguing brain-to-body metabolism and diabetes link was not intensely studied as the discovery of insulin in 1923 subsequently dominated the diabetes field. However, recent studies have provided important new insights into the role of the brain in the control of peripheral metabolic function and in the context of obesity-driven disorders, including type 2 diabetes. Accumulating evidence indicates that the brain closely monitors peripheral metabolic processes and plays a key role in regulating energy intake and metabolic homeostasis (Morton et al., 2006). The mechanisms involved in this regulation are complex, involving cholecystokinin, leptin, and insulin signaling, and several adiposity-related feedback loops (Morton et al., 2006; van Dijk et al., 2011).

Extensive research has characterized the hypothalamus as an important forebrain region in the regulation of metabolic homeostasis (van Dijk et al., 2011). Neuronal circuitry in the arcuate nucleus within the mediobasal hypothalamus plays a major regulatory role in food intake and metabolism mediating brain effects of leptin, insulin, melanocortins, and other metabolic molecules (Morton et al., 2006; Andermann and Lowell, 2017). For instance, the complex action of leptin on feeding behavior importantly involves activation of an appetite-suppressing population of pro-opiomelanocortin neurons and inhibition of neurons that express agouti-related protein and neuropeptide Y in the arcuate nucleus. Through a circuitry involving other proximal hypothalamic nuclei, such as the paraventricular nucleus and lateral hypothalamic area, these neurons communicate with the brainstem nucleus tractus solitarius (NTS) and promote afferent vagus nerve-mediated satiety and meal termination (Morton et al., 2006). Insulin also stimulates pro-opiomelanocortin neurons, which in turn causes suppression of appetite-promoting population of agouti-related protein neurons (Guilherme et al., 2019). In addition, insulin action in the arcuate nucleus is importantly implicated in the regulation of hepatic glucose production and adipose lipolysis (Konner et al., 2007; Shin et al., 2017). Specific insulin signaling in agouti-related peptide-expressing neurons mediates the suppression of hepatic glucose production by this molecule (Konner et al., 2007) while insulin action on pro-opiomelanocortin neurons is linked to restraining adipose tissue lipolysis (Shin et al., 2017).

**FIGURE 1:**
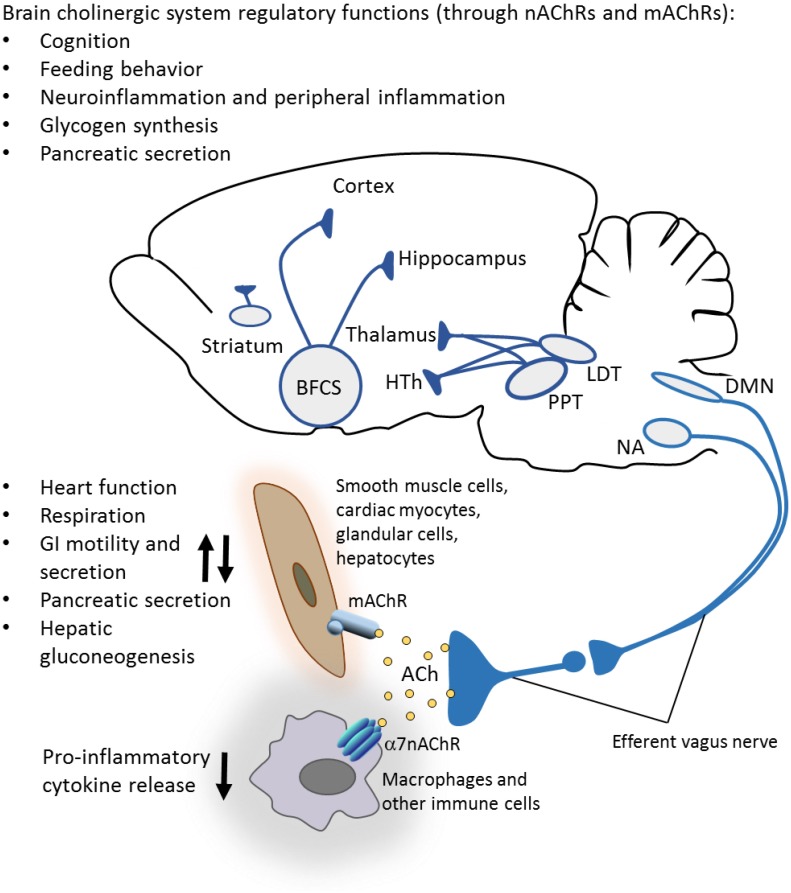
Brain cholinergic system: anatomy and functional control. Important constituents of the brain cholinergic system are the basal forebrain cholinergic system (BFCS) comprised of several nuclei, the mesopontine pedunculopontine and laterodorsal tegmental nuclei (PPT and LDT), and intraneurons in the striatum. Cholinergic neurons provide innervations to different cortex areas, hippocampus, amygdala, olfactory bulb, hypothalamus (HTh), thalamus, and other regions. In addition to its well-documented role in the regulation of cognition, brain cholinergic signaling is implicated in the regulation of appetite and feeding behavior, local brain inflammatory responses (neuroinflammation), hepatic glycogen synthesis, pancreatic secretion, and control of peripheral inflammation (through vagus nerve-mediated mechanisms). Cholinergic neurons in the brainstem dorsal motor nucleus of the vagus (DMN) and nucleus ambiguus (NA) provide axonal projections within preganglionic efferent vagus nerve fibers. These long fibers interact with short postganglionic neurons in proximity or within the innervated organs, including the heart, lungs, gastrointestinal tract, liver, and pancreas. Acetylcholine (ACh) released from these neurons interacts with muscarinic acetylcholine receptors (mAChRs) on targeted cells and regulates several metabolic functions. ACh also regulates (inhibits) the release of pro-inflammatory cytokines and inflammation via alpha 7 nicotinic acetylcholine receptors (α7nAChRs) on immune cells (see the text for more details). (A rodent brain is shown as a substantial part of the information summarized is based on preclinical studies.)

The pro-opiomelanocortin neuronal circuit in the hypothalamic arcuate nucleus was also identified as a major mediator of the appetite-suppressing effect of nicotine, thus providing a mechanistic insight into the apparent link between smoking and suppressed appetite (Mineur et al., 2011). α3β4nAChRs on these neurons have an essential role in mediating the suppressive effects of nicotine and other more selective agonists on food intake and weight gain in mice (Andermann and Lowell, 2017). These and other studies implicated brain α3β4 and other nAChRs, and the brain cholinergic system in the regulation of feeding behavior (Jo et al., 2002; Picciotto et al., 2012). As shown in Figure 1, major components of this system are the basal forebrain cholinergic system, comprised by several nuclei and the mesopontine/brainstem cholinergic system represented by the pedunculopontine and laterodorsal tegmental nuclei (Woolf and Butcher, 2011; Ballinger et al., 2016; Hampel et al., 2018). Basal forebrain cholinergic neurons innervate different cortex areas, the hippocampus, amygdala, and other regions (Figure 1). Among others, major projections of the cholinergic neurons in the mesopontine nuclei include the thalamus and hypothalamus (Figure 1). Cholinergic neurons residing in the brainstem dorsal motor nucleus of the vagus (DMN) and nucleus ambiguus (NA) provide peripheral axonal projections within the vagus nerve (Figure 1). Recently, basal forebrain cholinergic signaling was importantly implicated in the regulation of feeding behavior using selective optogenetic modulation (Herman et al., 2016; Figure 1). Both acute and chronic inactivation of cholinergic neurons in the basal forebrain diagonal band of Broca increases food intake while their stimulation results in decreased food intake (Herman et al., 2016). It was proposed that an extended brain network that regulates targets in the hypothalamic arcuate nucleus may mediate these cholinergic effects (Herman et al., 2016). In addition to nAChRs, muscarinic acetylcholine receptors (mAChRs) importantly mediate brain cholinergic neurotransmission and have also been associated with peripheral metabolic regulation. For instance, cholinergic mAChR-mediated activation in the hypothalamus results in increased hepatic glycogen synthesis and this effect is vagus nerve mediated (Shimazu et al., 1976; Matsushita et al., 1979). In addition, cholinergic M1 mAChR-mediated hypothalamic activation has also been shown to result in increased pancreatic exocrine secretion through the vagus nerve (Li et al., 2003; Figure 1).

**FIGURE 2:**
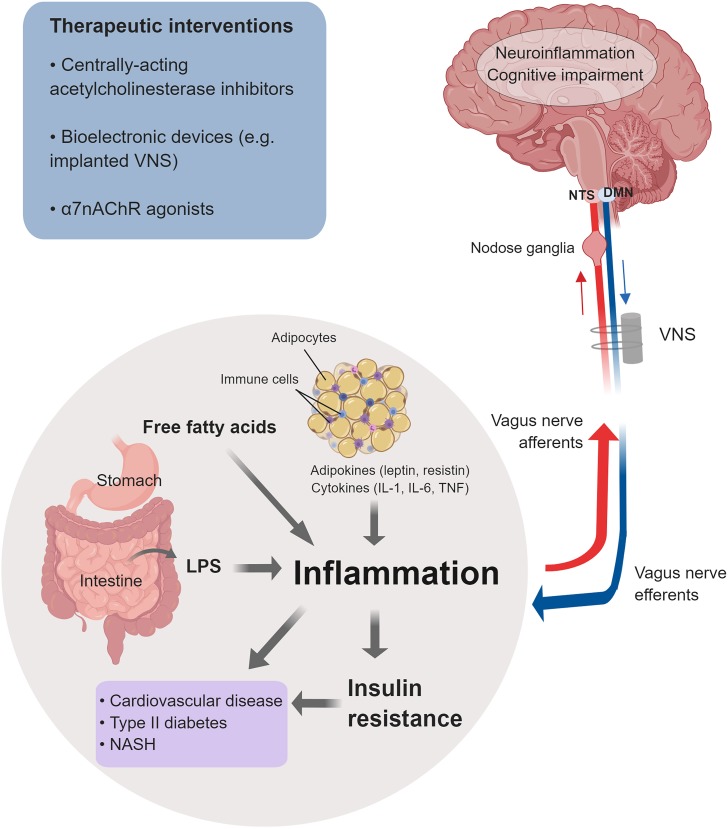
Cholinergic control of inflammation and metabolic derangements in obesity-driven disorders. Inflammation in obesity is a major driver of insulin resistance and other metabolic derangements linked to an increased risk of cardiovascular disease, type 2 diabetes, NASH, and other disorders. Main factors that contribute to this low-grade chronic inflammation are: adipokines and cytokines released from enlarged adipocytes and immune cells infiltrating the expanded white adipose tissue; LPS as a result of microbiota alterations and increased intestinal permeability; and increased levels of free fatty acids. Inflammation and neuroinflammation in obesity are also linked to cognitive impairment. The vagus nerve provides a major conduit for communication between the brain and the periphery. Afferent (sensory) neurons residing in the nodose ganglia and terminating in the NTS detect alterations in peripheral inflammatory and metabolic molecules and communicate this information to the brain. Signaling through efferent cholinergic fibers that originate in the dorsal motor nucleus (DMN) plays an important role in controlling inflammation and metabolic derangements. Brain cholinergic signaling also is implicated in the regulation of cognition and controlling neuroinflammation. Brain and peripheral (vagus nerve) cholinergic signaling can be explored for therapeutic benefit in obesity and obesity-driven conditions. Preclinical and clinical studies have shown the efficacy of galantamine and other centrally acting acetylcholinesterase inhibitors, bioelectronic VNS, and α7nAChRs agonists in alleviating inflammation, metabolic derangements, neuroinflammation, and in improving cognition (See text for details). (This figure was created with BioRender.)

The vagus nerve is a major conduit linking the brain and periphery in the regulation of metabolism (Berthoud, 2008; Andermann and Lowell, 2017; Metz and Pavlov, 2018). Sensory (afferent) fibers within the vagus nerve with cell bodies in the nodose ganglia communicate signals for alterations in nutrients and metabolic molecules, including cholecystokinin, leptin, and glucose from the gastrointestinal tract and the hepatic portal system to the brainstem (Pavlov and Tracey, 2012; Kaelberer et al., 2018; Figure 2). This communication involves neural synaptic transmission that occurs on the timescale of milliseconds, and slower humoral communication on the order of minutes. These signals arrive at NTS in the brainstem medulla oblongata, which is anatomically and functionally linked to DMN. Efferent (motor) vagus nerve cholinergic neurons originating from DMN and NA provide preganglionic innervations to visceral organs and regulate a range of vital cardiovascular, respiratory, and gastrointestinal functions, mediated through mAChRs on the effector cardiac myocytes, smooth muscle cells, and glandular cells (Figure 1). The efferent vagus nerve also regulates hepatic gluconeogenesis and pancreatic exocrine and endocrine secretion (Schwartz, 1983; Pocai et al., 2005; Pavlov and Tracey, 2012). For instance, efferent vagus nerve cholinergic signaling stimulates insulin release in the pancreas through M3 mAChR-mediated mechanism (Ruiz de Azua et al., 2011). In addition, mice with selective pancreatic β cells deficiency of the M3 mAChR have lower insulin secretion and impaired glucose tolerance (Gautam et al., 2006). NTS, DMN, and the closely located area postrema (a circumventricular organ) form the dorsal vagal complex, with reciprocal neuronal connectivity with hypothalamic nuclei and other forebrain regions, thus providing an extended brain network in the control of metabolic homeostasis (Pavlov and Tracey, 2012). The vagus nerve through its afferent fibers is a major mediator of satiety and a regulator of feeding behavior (Smith et al., 1981; Berthoud, 2008; Owyang and Heldsinger, 2011; Figure 2). Accordingly, a recent study demonstrated the substantial efficacy of implanted battery-free device-generated stimulation of afferent vagus nerve fibers (associated with stomach peristalsis) in achieving and maintaining weight loss in rats (Yao et al., 2018). This finding suggests the possibility of specifically targeting abdominal afferent vagus nerve fibers by bioelectronic devices as a therapeutic approach in obesity.

## Vagus Nerve and Brain Cholinergic Signaling in Controlling Inflammation

Inflammation is a vital physiological response to harmful stimuli, including pathogen invasion and tissue injury through a number of processes and pathways, including the activation of specific immune cell types (e.g., neutrophils and macrophages) and the release of inflammatory mediators (e.g., cytokines and chemokines) (Chen and Nunez, 2010; Olofsson et al., 2017). Inflammation is generally a localized event, which resolves and then the body returns to homeostasis (Chen and Nunez, 2010; Serhan and Levy, 2018). However, different forms of non-resolved, exacerbated, or chronic inflammation cause secondary tissue injury and mediate pathogenesis in sepsis, IBD, rheumatoid arthritis, and many other diseases (Firestein, 2003; Tracey, 2007; Chavan and Tracey, 2017). Therefore, controlling inflammation is critically important in disease prevention and a useful therapeutic strategy in disease treatment. In addition to immune and hormonal regulation, research during the last 20 years has demonstrated an important role of vagus nerve-mediated neural mechanisms in controlling inflammation (Chavan and Tracey, 2017). Several studies have shown that sensory vagus neurons can be activated by cytokines, including IL-1β, TNF, and other inflammatory molecules (Goehler et al., 2000; Steinberg et al., 2016; Zanos et al., 2018). These peripheral inflammatory alterations are communicated to the brainstem and in a reflex arc, vagus nerve cholinergic anti-inflammatory output is generated (Tracey, 2002).

**FIGURE 3:**
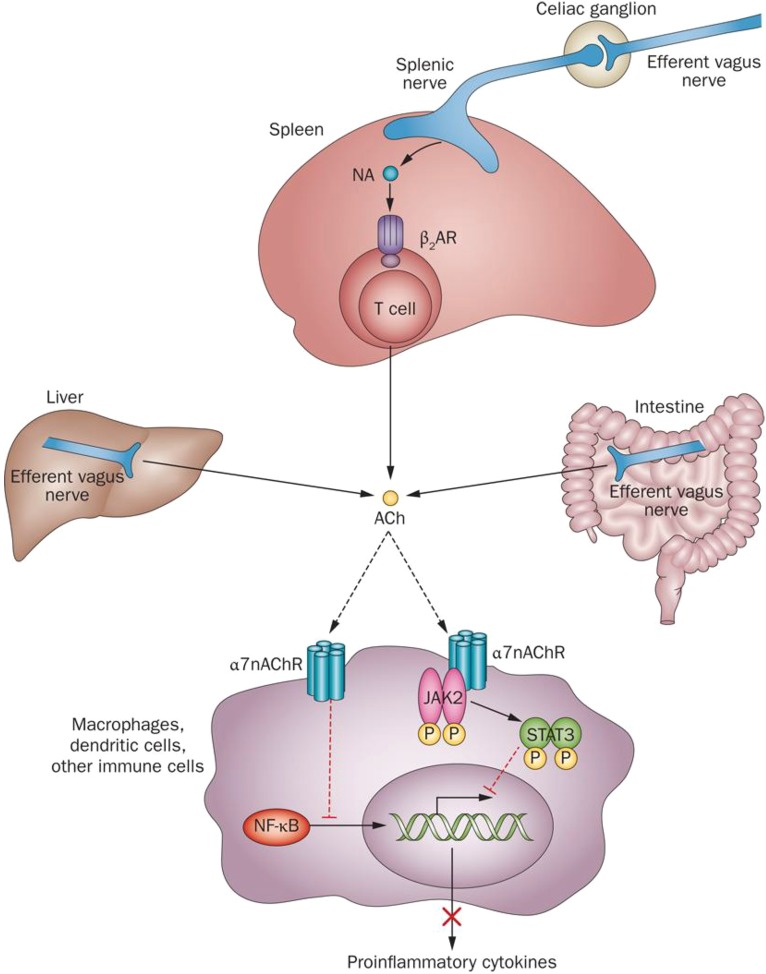
Molecular mechanisms of cholinergic control of inflammation. Efferent vagus nerve activity is translated into catecholamine-mediated activation of T-cell-derived ACh release in the spleen and into direct ACh release from efferent vagus nerve endings in other organs. Inhibition of NF-κB nuclear translocation and activation of a JAK2-STAT3-mediated signaling cascade in macrophages and other immune cells are implicated in cholinergic α7nAChR-mediated control of pro-inflammatory cytokine production. ACh, acetylcholine; β_2_AR, β_2_ adrenergic receptor; JAK2, Janus kinase 2; α7nAChR, α 7 nicotinic acetylcholine receptor; NA, noradrenaline; NF-κB, nuclear factor κB; STAT3, signal transducer and activator of transcription 3. (This figure was originally published in Nature Reviews Endocrinology. 2012; 8: 743–754 and is used here in agreement with Springer Nature copyright regulations for reuse of author’s own work.)

These studies led to the concept of a physiological immunoregulatory mechanism termed the inflammatory reflex (Tracey, 2002). The efferent arm of this mechanism was termed *the cholinergic anti-inflammatory pathway* (Borovikova et al., 2000; Pavlov et al., 2003). Electrical VNS has been instrumental in revealing the anti-inflammatory role of the vagus nerve innervating the liver, gastrointestinal tract, pancreas, and other organs in animal models (Borovikova et al., 2000; de Jonge et al., 2005; Bonaz et al., 2018; Metz and Pavlov, 2018). Cholinergic signaling is translated into suppression of pro-inflammatory cytokine release via α7nAChR-mediated signaling (Wang et al., 2003; Olofsson et al., 2012) and intracellular mechanisms, including suppression of NF-κB nuclear translocation, and JAK2/STAT3 activation (Figure 3) (Guarini et al., 2004; de Jonge et al., 2005; Parrish et al., 2008). In addition, recent studies have shown a mediating role for inflammasome inhibition and cAMP signaling (Tarnawski et al., 2018). Substantial advance in our understanding of the inflammatory reflex was achieved by revealing the functional cooperation between the vagus nerve and the splenic nerve and identifying a subset of splenic T cells, containing the enzyme choline acetyltransferase, as a source of acetylcholine in this circuit (Rosas-Ballina et al., 2011; Figure 3). Identifying the mediating role of the α7nAChR in the inflammatory reflex generated a line of research demonstrating the anti-inflammatory and disease-alleviating efficacy of α7nAChR agonists in numerous murine models of inflammatory diseases (Pavlov et al., 2007; Parrish et al., 2008; Pavlov and Tracey, 2015). Several studies have also shown that the inflammatory reflex and its efferent arm – the cholinergic anti-inflammatory pathway – can be activated through brain mAChR signaling. The anti-inflammatory and beneficial metabolic effects of centrally acting mAChR ligands and the AChE inhibitor galantamine have been demonstrated in murine models of endotoxemia, IBD, hemorrhagic shock, lupus, and other disorders and linked to the inflammatory reflex (Pavlov et al., 2006, 2009; Lee et al., 2010; Ji et al., 2014; Munyaka et al., 2014; Rosas-Ballina et al., 2015; Pham et al., 2018). In addition to galantamine, the anti-inflammatory effects of other AChE inhibitors and cholinergic drugs clinically approved for the treatment of Alzheimer’s disease, such as donepezil and rivastigmine, have also been demonstrated (Lataro et al., 2015; Pavlov and Tracey, 2015; Zhang et al., 2016).

In addition to inflammation in the periphery, inflammation in the CNS and specifically in the brain also occurs in response to tissue damage and pathogens. Persistent neuroinflammation is a characteristic feature of traumatic brain injury, sepsis, multiple sclerosis, other neurodegenerative diseases, and other disorders (Amor et al., 2014; Borst et al., 2018; Pavlov et al., 2018). A link between inflammation, neuroinflammation, and cognitive deterioration has also been identified (Nizri et al., 2008; Terrando et al., 2011; Miller and Spencer, 2014; McManus and Heneka, 2017; Borst et al., 2018). Of note, galantamine, rivastigmine, and donepezil have been shown to alleviate neuroinflammation and improve cognition in preclinical studies (Nizri et al., 2008; Dasuri et al., 2016; Wang et al., 2018; Figure 2). In addition, a very recent study demonstrated that in addition to suppressing peripheral inflammation, VNS also alleviates neuroinflammation and cognitive dysfunction in murine endotoxemia (Huffman et al., 2019).

## Cholinergic Control of Obesity-Associated Inflammation and Metabolic Derangements

Chronic low-grade inflammation is a characteristic pathological feature of obesity and MetS (Eckel et al., 2005; Tilg and Moschen, 2006; Nathan, 2008; Gregor and Hotamisligil, 2011; Lumeng and Saltiel, 2011; Pavlov and Tracey, 2012). Inflammation in obesity is manifested by increased circulating levels of classical pro-inflammatory cytokines, such as TNF and altered levels of adipokines, including leptin, resistin, and adiponectin (Tilg and Moschen, 2006; Lumeng and Saltiel, 2011; Pavlov and Tracey, 2012). Expanded abdominal white adipose tissue in obesity and the crosstalk between metabolically active adipocytes and immune cells infiltrating the adipose tissue, including macrophages, neutrophils, and T lymphocytes, have been identified as a major source of cytokines and adipokines and contributors to this characteristic inflammation (Tilg and Moschen, 2006; Nishimura et al., 2009; Pavlov and Tracey, 2012; Engin, 2017; Figure 2). Both enlarged adipocytes and infiltrating immune cells release pro-inflammatory cytokines such as TNF, interleukin-1 (IL-1β), and IL-6 (Pavlov and Tracey, 2012; Engin, 2017). Increased circulating levels of lipopolysaccharide (LPS, endotoxin) have been also detected in obesity (Cani et al., 2007). Microbiota alterations in the gut (increased LPS-containing microbiota) as a result of high-fat diet intake and increased body weight, and the consequent increased intestinal permeability have been associated with this “metabolic endotoxemia,” which is another major contributor to inflammation in obesity (Cani et al., 2007; Cani and Delzenne, 2009; Delzenne et al., 2011). LPS, acting through a toll-like receptor 4 (TLR4)-mediated mechanism, triggers the release of TNF and other pro-inflammatory cytokines, mediating pro-inflammatory signals in liver, skeletal muscle, and adipose tissue (Cani et al., 2007; Castanon et al., 2014). Another important contributor to inflammation and other metabolic derangements in obesity is the high levels of free fatty acids (Lumeng and Saltiel, 2011). Acting through TLR4-mediated mechanisms on adipocytes, macrophages, and hepatocytes, free fatty acids trigger intracellular signaling resulting in NF-κB activation and increased TNF and other pro-inflammatory cytokine release (Shi et al., 2006; Baker et al., 2011; Lumeng and Saltiel, 2011; Pavlov and Tracey, 2012). Inflammation in obesity is linked to insulin resistance (Cani et al., 2007; Shoelson et al., 2007; Olefsky and Glass, 2010; Vandanmagsar et al., 2011). For instance, TNF has been shown to directly induce insulin resistance (Hotamisligil et al., 1993; Hotamisligil et al., 1996). In addition, obesity-related inflammation and insulin resistance are linked to fatty liver disease and the development of non-alcoholic steatohepatitis (NASH) (Shoelson et al., 2007; Carter-Kent et al., 2008; Lumeng and Saltiel, 2011; Schuppan and Schattenberg, 2013; Figure 2).

Approaches based on electrical VNS have been successfully explored in the treatment of obesity (Val-Laillet et al., 2010; Malbert et al., 2017; Masi et al., 2018). Several studies have also indicated the efficacy of targeting the inflammatory reflex using pharmacological modalities in alleviating inflammation and metabolic derangements interrelated in obesity-driven conditions (Pavlov and Tracey, 2012; Figure 2). Administration of nicotine (an α7nAChR agonist) to genetically obese *db/db* mice lacking the leptin receptor and mice with high-fat diet-induced obesity suppresses adipose tissue and systemic TNF levels (Wang et al., 2011). Nicotine also decreases adipose tissue expression of *CCL2* and the macrophage marker *F4/80*, pointing to alleviation of adipose tissue macrophage infiltration. In addition, α7nAChR KO mice on a high-fat diet have increased M1 macrophage infiltration and upregulated expression of TNF and CCL2 in adipose tissue compared to WT controls (Wang et al., 2011). Oral administration of the selective α7nAChR agonist TC-7020 to *db/db* mice significantly lowers systemic TNF and this effect is abrogated by co-administering methyllycaconitine – a selective α7nAChR antagonist (Marrero et al., 2010). TC-7020 administration to *db/db* mice also reduces weight gain, food intake and blood glucose, HbA1c, and triglyceride levels and these beneficial effects are also abrogated by methyllycaconitine (Marrero et al., 2010). Importantly, administration of a JAK2 inhibitor significantly diminishes the TC-7020 effects on body weight, food intake, and blood glucose levels, a finding that suggests a link between α7nAChR- and JAK2-mediated signaling (Marrero et al., 2010). An important role for vagus nerve α7nAChR-mediated cholinergic signaling in experimental NASH was also demonstrated (Nishio et al., 2017). In a murine model of diet-induced NASH, α7nAChR KO chimeric mice (produced by transplanting α7nAChR bone marrow cells into γ-irradiated and Kupffer cell-depleted wild-type recipients) develop an accelerated form of disease (Nishio et al., 2017). These mice have significantly upregulated pro-inflammatory cytokine expression and altered/abnormal lipid metabolic pattern. Selective hepatic vagotomy in this model also results in increased TNF, IL-12, and CCL2 (MCP-1) levels indicative for increased hepatic inflammation (Nishio et al., 2017). Using α7nAChR KO mice, another recent study also demonstrated the tonic anti-inflammatory and anti-fibrotic role of this receptor in models of atherogenic high-fat diet- and methionine/choline-deficient diet-induced NASH (Kimura et al., 2018). In these models, α7nAChR deficiency resulted in exacerbated hepatic fibrosis, higher plasma transaminase levels, and significantly increased Col1a1 gene-encoding alpha-1 type I collagen (mediating liver fibrosis) *Ccl2* and *Tnf* gene expression (Kimura et al., 2018).

As noted above, the inflammatory reflex can be activated by the centrally acting cholinergic drug, the AChE inhibitor galantamine (Pavlov et al., 2009; Ji et al., 2014; Pham et al., 2018). Galantamine alleviates inflammation and metabolic derangements in a high-fat diet-induced model of obesity and MetS (Satapathy et al., 2011). Galantamine treatment of mice with established obesity (after 8 weeks on a high-fat diet) significantly lowers plasma IL-6, CCL2, leptin, and resistin levels, and reduces body weight, food intake, and abdominal white adipose depots (Satapathy et al., 2011). Galantamine also decreases blood glucose, plasma insulin, and cholesterol levels, and alleviates insulin resistance and fatty liver disease in these mice (Satapathy et al., 2011). Recent work has also shown that galantamine has anti-diabetic effects in murine models (Ali et al., 2015; Hanes et al., 2015). Of note, the anti-diabetic effects of galantamine treatment of rats with established n5-STZ diabetes are greater than the effects of the anti-diabetic drug vildagliptin also used in the study (Ali et al., 2015). These and other preclinical studies indicate that neural cholinergic modulation, either pharmacologically or through bioelectronic VNS, can be further explored to treat obesity-related inflammatory and metabolic derangements.

## Neuroinflammation and Cognitive Impairment in Obesity-Driven Disorders: a Cholinergic Link to Treatment

In addition to inflammation in the periphery, obesity is associated with neuroinflammation (Miller and Spencer, 2014; Guillemot-Legris and Muccioli, 2017; Lainez et al., 2018; Figure 2). This neuroinflammation occurs in multiple brain structures, including the hypothalamus, hippocampus, amygdala, neocortex, and cerebellum, and there is evidence that it is sex-specific (Miller and Spencer, 2014; Guillemot-Legris and Muccioli, 2017; Lainez et al., 2018). In these brain regions, diet-induced obesity is associated with increased levels of pro-inflammatory cytokines along with higher expression of NF-κB and TLR4, two important molecular mediators of innate immune responses (Biessels et al., 2014). This type of neuroinflammation may involve recruitment of peripheral immune cells (Miller and Spencer, 2014; Lainez et al., 2018) and there is evidence that peripheral inflammation precipitates brain inflammation (Miller and Spencer, 2014; Guillemot-Legris and Muccioli, 2017). Studies have linked obesity with cognitive impairment and both inflammation and neuroinflammation may play a mediating role in this context (Pistell et al., 2010; Sellbom and Gunstad, 2012; Miller and Spencer, 2014). Association between obesity and diabetes derangements, and increased risk of developing dementia has also been indicated (Whitmer et al., 2005; Strachan et al., 2011; Biessels et al., 2014).

The brain cholinergic system (Figure 1) plays a major role in the regulation of memory, attention, and learning (Ballinger et al., 2016; Hampel et al., 2018). Cholinergic neurodegeneration predominantly affecting the basal forebrain neurons and neuroinflammation are hallmarks of brain pathology in Alzheimer’s disease (Heneka et al., 2015; Hampel et al., 2018). As mentioned earlier, centrally acting AChE inhibitors, including galantamine, donepezil, and rivastigmine, are clinically approved cholinergic drugs for treating cognitive impairment in Alzheimer’s disease (Hampel et al., 2018). There is accumulating experimental evidence that brain cholinergic dysfunction is implicated in cognitive impairment in obesity as recently reviewed (Martinelli et al., 2017). High-fat diet-induced obesity in mice results in increased brain AChE expression and neuroinflammation manifested by microglial activation (Dasuri et al., 2016). Of note, donepezil treatment suppresses brain microglial activation and neuroinflammation in this model (Dasuri et al., 2016). There is also experimental evidence that enhancing cholinergic signaling by galantamine and rivastigmine also results in anti-inflammatory effects in the brain and improved cognition (Nizri et al., 2008; Wang et al., 2018). It is interesting and important that many brain regions affected by neuroinflammation in obesity, including the hypothalamus, hippocampus, amygdala, and cortex receive cholinergic innervations (Figure 1). Whether cholinergic dysfunction facilitates neuroinflammation in obesity is a question that remains to be further addressed.

Together these findings paint a picture of peripheral inflammation and neuroinflammation interrelated with cognitive impairment and cholinergic dysfunction in obesity. An improved understanding of the relationship between these elements of pathology in obesity would provide a solid rationale for designing new therapeutic approaches.

## Clinical Translation of Cholinergic Modulation in Obesity-Driven Disorders

Obesity and obesity-related conditions, including MetS, type 2 diabetes, NASH, and cardiovascular disease, present a substantial health burden. Current treatment options are limited to lifestyle modifications, non-specific drugs, or insulin injections for those with type 2 diabetes. Unfortunately, lifestyle changes and dietary modifications are only temporarily effective for durable weight loss. For instance, a study with obese women subjected to caloric restrictions and behavioral therapy showed that nearly 50% of lost weight is regained within 1 year and almost all of it is regained within 5 years (Wadden et al., 1989). Non-specific weight loss medications have a blemished track record and are also often ineffective as long-term solutions. Obesity-related type 2 diabetes is a progressive disorder and most patients eventually require treatment with insulin to control blood glucose levels as β cells in the pancreas lose their ability to produce insulin (Kahn and Hull, 2006). While generally effective, injectable insulin can produce side effects such as hypoglycemia, hunger, weakness, irritability, and requires frequent monitoring of blood glucose levels. The current studies on closed-loop control systems in diabetes known as “the artificial pancreas” may provide a significant advance in overcoming these disadvantages (Kovatchev, 2018). Other treatment options for obesity-driven disorders include surgical interventions with bariatric surgery which can result in long-term weight loss, but surgical treatments come with significant risks and are only recommended for the morbidly obese (Chang et al., 2014). Thus, the limitations of currently available treatments for obesity-driven disorders invite an exploration of new targets and pathways. In this context, targeting inflammation remains an attractive area for further exploration (Goldfine et al., 2011; Esser et al., 2015).

Based on preclinical evidence for beneficial anti-inflammatory and metabolic efficacy, cholinergic modulation in treating obesity-driven disorders is of specific interest (Figure 2). An additional advantage is that cholinergic drugs such as centrally acting AChE inhibitors and bioelectronics, including implanted devices for VNS are already clinically approved for other indications. Galantamine is a centrally acting AChE inhibitor that has been FDA approved to treat the cognitive impairment in patients with Alzheimer’s disease in the United States for more than a decade (Hampel et al., 2018). There is a large amount of safety information available, making galantamine an attractive candidate to be clinically repurposed. A major obesity-driven disorder is MetS, a cluster of conditions that includes high blood pressure, high blood glucose levels, abdominal obesity, and dyslipidemia (Eckel et al., 2005; Grundy, 2008). The combination of these conditions within MetS generates a significantly higher risk of developing type 2 diabetes, cardiovascular disease, cancer, and other life-threatening and debilitating diseases, compared to the individual condition-related risk (Eckel et al., 2005). Apart from lifestyle modifications, treating MetS as a whole presents a significant challenge, and usually several medications targeting the separate risk factors are prescribed. Inflammation as a driver of insulin resistance and other pathogenesis in MetS provides an attractive therapeutic target (Esser et al., 2015). Based on preclinical findings, the efficacy of galantamine in alleviating inflammation and insulin resistance alongside other metabolic indices in people with MetS was recently studied in a randomized, placebo-controlled, double blind trial (Consolim-Colombo et al., 2017). Relatively short treatment (for 12 weeks) with galantamine in doses clinically approved for Alzheimer’s disease, significantly decreased plasma TNF and leptin levels and increased IL-10 and adiponectin levels (Consolim-Colombo et al., 2017). Galantamine treatment vs. placebo also modulated the autonomic neural regulation, as determined by heart rate variability analysis, toward parasympathetic (vagal) predominance (Consolim-Colombo et al., 2017).

Another approach of cholinergic neuromodulation to explore in treating obesity-driven diseases is VNS. The feasibility of this approach for treating immune and metabolic derangements in humans was recently demonstrated in clinical trials with patients with IBD and rheumatoid arthritis (Bonaz et al., 2016; Koopman et al., 2016). Interestingly, humans with obesity have reduced vagal tone (Karason et al., 1999; Carnethon et al., 2003), coexisting with inflammation and metabolic deterioration. Since activation of vagal efferents attenuates immune and metabolic dysfunction, it is possible that reduced vagal cholinergic output plays a causative role in immune and metabolic dysregulation. VNS is clinically approved for epilepsy and depression (Ben-Menachem, 2001; Bonaz, 2018). Of note, patients receiving VNS for medication-refractory epilepsy or depression have also reported weight loss, in as high as 60% of the patients tested (Burneo et al., 2002; Pardo et al., 2007). While these patients were receiving VNS for other non-metabolic indications, these weight loss effects suggest the possible therapeutic utility of VNS in obesity-associated conditions. VNS implants for epilepsy and depression have been routinely used and the therapy is well-tolerated (Ben-Menachem, 2001). The risks associated with VNS implantation surgery are low and side effects of the stimulation itself are minor, typically limited to cough and hoarseness (Sackeim et al., 2001; O’Reardon et al., 2006). A recent study demonstrated that specific vagus nerve signals in response to alterations in cytokine levels can be recorded (Zanos et al., 2018). Therefore, in the future, an advanced version of bioelectronic vagus nerve modulation could potentially provide the additional advantage of enabling “closed-loop” control of immune and metabolic dysfunctions. Preclinical research has also indicated the possibility to use α7nAChR agonists in treating inflammatory and metabolic derangements in human obesity. This is also supported by demonstrating α7nAChR expression in human adipocytes and its role in controlling pro-inflammatory gene expression (Cancello et al., 2012). Furthermore, obesity is associated with significant decrease in human adipocyte α7nAChR and weight loss partially restores its expression (Cancello et al., 2012).

Cognitive deterioration and an increased risk of developing dementia are documented in obesity, MetS, and type 2 diabetes (Strachan et al., 2011; Biessels et al., 2014). This cognitive dysfunction is interrelated with inflammatory and metabolic derangements and treating these conditions is challenging (Strachan et al., 2011; Guillemot-Legris and Muccioli, 2017). An important point is that while designing new approaches for treating obesity and related conditions, one should account for their effects on the brain in relation to cognitive impairment (Strachan et al., 2011). The cholinergic system in the brain has a key role in the regulation of cognition (Picciotto et al., 2012; Ballinger et al., 2016). Numerous studies have evaluated galantamine, donepezil, and rivastigmine as cognitive enhancers and these drugs are in current clinical use for the symptomatic treatment of Alzheimer’s disease. As recently demonstrated, galantamine has beneficial anti-inflammatory and metabolic effects in patients with MetS (Consolim-Colombo et al., 2017). Therefore, future developments with galantamine and other centrally acting AChE inhibitors in the treatment of patients with obesity-associated disorders may include assessing the potential benefit of these drugs on cognition. VNS has been also associated with pro-cognitive effects especially in the domains of verbal recognition, memory, and executive function (Groves and Brown, 2005; Vonck et al., 2014). The precise mechanisms of these VNS pro-cognitive effects are largely unknown and one possibility that remains to be explored is a mediating role of brain cholinergic signaling. Recent findings have indicated that NTS afferent vagus nerve signaling reaches basal forebrain cholinergic nuclei innervating the hippocampus and cortex and implicated in cognitive regulation (Suarez et al., 2018). These studies and other clinical trials currently underway (ClinicalTrials.gov Identifier: NCT02365285) generate a growing platform for further studies on the therapeutic utility of cholinergic modulation in obesity-associated disorders.

## Concluding Remarks

In obesity and obesity-associated conditions, immune and metabolic dysregulation result in chronic systemic inflammation, neuroinflammation, exacerbated insulin resistance, fatty liver disease, cognitive impairment, and other pathological manifestations. An improved understanding of this complex pathology requires providing new insight into the regulatory role of the nervous system. Neural circuitry, including vagus nerve cholinergic signaling, plays a major role in controlling metabolic and immune homeostasis (Figure 1–3). Vagus nerve cholinergic signaling within the inflammatory reflex has an important regulatory role in the crosstalk between immune and metabolic alterations in obesity-driven disorders. Activation of cholinergic signaling by VNS, α7nAChR agonists, and centrally acting drugs such as galantamine results in anti-inflammatory effects, alleviation of insulin resistance and hepatic steatosis, and other beneficial effects in murine models of obesity, MetS, NASH, and type 2 diabetes. This large body of preclinical evidence and the fact that both centrally acting AChE inhibitors and VNS are already in clinical use provide a rationale for expanding these approaches into clinical settings of obesity. A recent clinical trial with galantamine in MetS demonstrated the translational applicability of this research and the anti-inflammatory and beneficial metabolic effects of AChE inhibitors (Consolim-Colombo et al., 2017). Enhancing brain cholinergic signaling by these drugs and the use of VNS to alleviate obesity-associated brain pathology, including neuroinflammation and cognitive deterioration in humans, are feasible approaches that remain to be studied. Recent discoveries of gut–brain neural circuits that involve the vagus nerve (Han et al., 2018; Kaelberer et al., 2018) and future preclinical research using advances in molecular genetics within the growing field of bioelectronic medicine (Olofsson and Tracey, 2017; Pavlov and Tracey, 2019) will improve our understanding of neural regulation of immunity and metabolism and its implications in obesity. This research holds the potential to identify new therapeutic avenues for alleviation of the obesity disease burden.

## Author Contributions

EHC, SSC, and VAP wrote, edited, and finalized the manuscript.

## Conflict of Interest Statement

SC and VP are authors on patents broadly related to the content of this review and have assigned their rights to the Feinstein Institute for Medical Research. The remaining author declares that the research was conducted in the absence of any commercial or financial relationships that could be construed as a potential conflict of interest.
